# Disparities between Ophthalmologists and Patients in Estimating Quality of Life Associated with Diabetic Retinopathy

**DOI:** 10.1371/journal.pone.0143678

**Published:** 2015-12-02

**Authors:** Xiaofeng Zhu, Qian Sun, Haidong Zou, Xun Xu, Xi Zhang

**Affiliations:** Department of Ophthalmology, Shanghai First People's Hospital, affiliated Shanghai Jiaotong University, Shanghai, China; Medical College of Soochow University, CHINA

## Abstract

**Background:**

This study aimed to evaluate and compare the utility values associated with diabetic retinopathy (DR) in a sample of Chinese patients and ophthalmologists.

**Methods:**

Utility values were evaluated by both the time trade-off (TTO) and rating scale (RS) methods for 109 eligible patients with DR and 2 experienced ophthalmologists. Patients were stratified by Snellen best-corrected visual acuity (BCVA) in the better-seeing eye. The correlations between the utility values and general vision-related health status measures were analyzed. These utility values were compared with data from two other studies.

**Results:**

The mean utility values elicited from the patients themselves with the TTO (0.81; SD 0.10) and RS (0.81; SD 0.11) methods were both statistically lower than the mean utility values assessed by ophthalmologists. Significant predictors of patients’ TTO and RS utility values were both LogMAR BCVA in the affected eye and average weighted LogMAR BCVA. DR grade and duration of visual dysfunction were also variables that significantly predicted patients’ TTO utility values. For ophthalmologists, patients’ LogMAR BCVA in the affected eye and in the better eye were the variables that significantly predicted both the TTO and RS utility values. Patients’ education level was also a variable that significantly predicted RS utility values. Moreover, both diabetic macular edema and employment status were significant predictors of TTO and RS utility values, whether from patients or ophthalmologists. There was no difference in mean TTO utility values compared to our American and Canadian patients.

**Conclusions:**

DR caused a substantial decrease in Chinese patients’ utility values, and ophthalmologists substantially underestimated its effect on patient quality of life.

## Introduction

Diabetic retinopathy (DR) is the major cause of acquired vision loss and is the most common microvascular complication of diabetes [1]. With rapid lifestyle changes occurring in China, the estimated prevalence of diabetes has increased to 11.6% among Chinese adults [2], with at least 20% of diabetic patients suffering from DR [3–5]. Visual impairment from DR places a considerable burden on patients’ quality of life (QoL) [6–10].

In recent years, the QoL of DR patients has gradually become a concern among ophthalmologists. A variety of vision-specific functioning and QoL questionnaires, such as the Visual Functioning Index (VF-14) [11], the National Eye Institute’s Visual Function Questionnaire (NEI-VFQ-51/-25) [12,13], and the Impact of Vision Impairment (IVI) questionnaire [14–16], offer tools for studying the QoL of DR patients. Outcome analyses of the questionnaires showed that the QoL of DR patients was significantly lower than that of healthy individuals. Nevertheless, these questionnaires have shortcomings, including a limited number of questions and the incomplete assessment of DR patients’ QoL, their subjective desires and perceptions.

Utility measures of health-related QoL are preference values that patients attach to their overall health status [17]. Conventionally, a utility value is a value between two extreme endpoints, 1.0 and 0.0, where 1.0 implies a perfect health state and 0.0 usually signifies death [18,19]. A higher utility value reflects a higher patient QoL, including capacities for physical activities and social and psychological health [20]. Utility values for DR, using both the Standard Gamble (SG) and time trade-off (TTO) methods, have been applied in several studies. However, the overall utility values have considerable variability, which is likely caused by differences in study design, methodology, sample size, and DR severity, with or without diabetic macular edema (DME).

After reviewing the English and Chinese literature, using PubMed and Chinese BioMedical Literature (CBM) databases, we found that only one study assessed the utility values associated with DR in a non-Western population [21]. However, China has the greatest public health burden of DR in the world, and DR patients’ QoL relates to their culture and geography [2]. Furthermore, Brown suggested that it is important to appreciate the disparity in estimations of utility values between age-related macular degeneration (AMD) patients and ophthalmologists [22], and ophthalmologists should incorporate the needs and wants of the patient into treatment decisions. To our knowledge, no published report has shown the disparities between patients’ and ophthalmologists’ perceptions of QoL associated with DR.

The present study was performed to evaluate the utility values associated with DR in a Chinese population using the rating scale (RS), another commonly used method for utility values, and the TTO method to compare the assessed patient and ophthalmologist utility values and to demonstrate some related indexes of these utility values. In addition, we investigated the consistency of utility values among patients with DR through a comparison of our sample and the other two samples obtained in a similar manner in different countries.

## Materials and Methods

### Study design and population

In this cross-sectional study, consecutive patients were recruited from a predominantly vitreoretinal and comprehensive ophthalmologic outpatient service at the First People’s Hospital affiliated to Shanghai Jiaotong University, China, between October 2014 and March 2015.

The main inclusion criteria were that patients had a diagnosis of DR, at least a history of diabetes mellitus (DM) associated with retinal microaneurysms, and had been suffering from visual impairment (20/30 or worse in at least one eye). The latter was defined as either visual impairment occurring primarily secondary to DR or the exclusion of primary visual impairment caused by other reasons (such as cataract, glaucoma, or AMD), which have been previously reported [23]. Patients were excluded from the study for inability or unwillingness to answer the questions posed. In addition, patients with Alzheimer’s disease or other forms of dementia who were poorly communicative were also excluded.

This study was approved by the institutional review boards of the First People’s Hospital affiliated to Shanghai Jiaotong University and adhered to the tenets of the Declaration of Helsinki. Moreover, written informed consent was obtained from all study patients.

All patients underwent a comprehensive ophthalmologic examination that included determining the Snellen best corrected visual acuity (BCVA) in both eyes, an anterior segment examination, dilated funduscopy, and an assessment of the DR grade [24] (according to the annual meeting of the American Academy of Ophthalmology proposed international clinical disease severity grading scale for DR in 2002) and the presence of DME.

A standardized interview was performed by experienced researchers trained in utility valuation. Detailed demographic information including age, gender, years of formal education after kindergarten, employment status, marital status, the number of systemic co-morbidities (including cardiac, respiratory, neurologic diseases, and cancer), the duration of DM (time since the onset of DM diagnosis), and the duration of visual dysfunction were collected in the interview. The TTO and RS methods for calculating the utility values were used to evaluate the patients’ health-related QoL.

TTO visual utility values from patients were measured using a standard methodology described in other DR studies [23,25–28]. Patients were asked two hypothetical questions: (1) how many years of remaining life they expect to live; and (2) in patients with abnormal visual acuity (<20/30 in at least one eye), each was asked to quantify the maximum number of years of remaining life, if any, they would be willing to trade in return for permanently perfect vision in each eye. In patients with good bilateral visual acuity (20/20-20/25), each was asked to quantify the maximum number of years of their remaining life, if any, that they would be willing to trade in return for a guarantee of retaining good vision in each eye for the remaining years. The utility value was calculated by these two pieces of data as follows: utility value = 1.0 –X (X = time traded/time of remaining life). For example, if a 60-year-old patient expects to live 20 years and would be willing to trade in return 5 years for perfect vision, the utility value is calculated as 1.0−5/20 = 0.75.

The RS was a vertical and calibrated visual analogue scale (0–100). The patients were asked the subject question: On a scale where 0 represents blind and 100 represents perfect vision, where would you rate your current vision? The score (Q) was chosen by patients, and the data obtained were used to calculate the following: utility value = Q/100.

Ophthalmologists were asked to assume they had the same health status as each corresponding patient and then to assess utility values according to his or her own perceptions. The two ophthalmologists, with an average age of 40 and each having more than 10 years’ of experience in vitreoretinal diseases, made their final assessments. Ophthalmologists who traded more time or chose a lower scale than did actual patients (as indicated by lower utility values) may have overestimated the impact that a medical condition has on QoL. Conversely, ophthalmologists who opted to give up less time or chose a higher scale (as indicated by higher utility values) may have underestimated patients’ suffering. The utility values assessment was double-masked so that neither the patient nor the ophthalmologist had access to the other’s utility values.

### Data management and analysis

Before the study was undertaken, sample size was calculated employing values from a previous study [23] with SPSS Sample Power 3.0 (SPSS Inc., Chicago, IL, USA), with a 2-sided alpha of 0.05 and 90% power. A total of 94 patients was necessary to demonstrate a 10% difference in mean utility values, and a total of 43 patients was necessary to demonstrate a 15% difference.

Snellen BCVA was converted to the logarithm of the minimum angle of resolution (LogMAR) for statistical analysis [29]. The weighted average LogMAR BCVA of both eyes was calculated as follows: the weighted average gave a 0.75 weighting to the better eye and a 0.25 weighting to the worse eye [30].

Descriptive statistical analyses were performed to characterize the demographic data, visual acuity, clinical characteristics, and utility values. The paired, 2-tailed Student t test was used to compare the mean utility values of the TTO and RS between patients and ophthalmologists. Box-plots were used to provide a more detailed distribution of the utility values. Correlations of the utility values from patients and ophthalmologists were analyzed with linear regression analysis. A correlation coefficient (R^2^) ≥ 0.70 was considered to be a significant correlation. The independent-samples t test and the Mantel-Haenszel chi-square test were used to compare the major clinical characteristics of the patients.

Multivariate linear regression was used to evaluate the relationship of the utility values, whether from DR patients or ophthalmologists, and the independent variables of age, education, the number of systemic comorbidities, the duration of DM and visual dysfunction, DR grade, LogMAR BCVA in affected eye, LogMAR BCVA in the better-seeing eye, and the weighted average LogMAR BCVA at the same time point, using an entry *p* = 0.05 and an exit *p* = 0.10. Bivariate analyses were performed to determine the association between the utility values and the dichotomous variables of interest (gender, DME, employment and marital status). Pearson correlation coefficients and analysis of variance (ANOVA) were used with appropriate significance tests. One-way ANOVA and the Dunnett t test were used to compare our TTO data with those of Brown and associates [23] obtained from 95 American patients with DR and those of Sharma and associates [28] obtained from 186 Canadian patients with DR.

The study patients were divided into five groups according to the Snellen BCVA (LogMAR BCVA) in the better-seeing eye: Group 1, 20/20 to 20/25 (1.0 to 0.8); Group 2, 20/30 to 20/50 (0.6 to 0.4); Group 3, 20/60 to 20/100 (0.3 to 0.2); Group 4, 20/200 to 20/400 (0.1 to 0.05); and Group 5, worse than 20/400 (<0.05).

The data were analyzed using SPSS statistical software Version 13.0 (SPSS Inc., Chicago, IL, USA). An alpha level of *p* < 0.05 was chosen as the criterion for significance.

## Results

A total of 120 patients were screened for the study; however, 11 were excluded because of their inability to answer the questions. Thus, data from 109 (90.8%) patients with DR were included. Patient demographic and clinical characteristics are shown in Table 1.

**Table 1:** Demographic and clinical characteristics of 109 patients with diabetic retinopathy.

Characteristic	109 patients with DR
Mean age (SD, 95%CI) years	50.6 (10.2, 95%CI, 48.70 to 52.55)
Male [No. (%)]	58 (53.2)
Mean education (SD, 95%CI) years	13.5 (5.0, 95% CI, 12.57 to 14.48)
Less than 12 years (less than high school) [No. (%)]	33 (30.3)
12 years (high school) [No. (%)]	20 (18.3)
More than 12 years (beyond high school) [No. (%)]	56 (51.4)
Marital Status	
Married/common-law [No. (%)]	72 (67.0)
Single, widowed, divorced, and separated [No. (%)]	36 (33.0)
Employment Status	
Employed [No. (%)]	68 (62.4)
Retired, never worked, and disabled or looking for work [No. (%)]	41 (37.6)
No. of systemic comorbidities	
0 [No. (%)]	25 (22.9)
1 [No. (%)]	46 (42.2)
2 [No. (%)]	24 (22.0)
≥3 [No. (%)]	14 (12.8)
Mean LogMAR BCVA in affected eye (SD, 95%CI)	0.65 (0.40, 95% CI, 0.57 to 0.73)
Mean LogMAR BCVA in the better eye (SD, 95%CI)	0.17 (0.29, 95% CI, 0.11 to 0.22)
Average weighted LogMAR BCVA (SD, 95%CI)	0.29 (0.28, 95% CI, 0.23 to 0.34)
Mean duration of DM (SD, 95%CI) yrs	5.49 (5.18, 95% CI, 4.50 to 6.47)
Mean duration of visual dysfunction (SD, 95%CI) weeks	1.72 (0.99, 95% CI, 1.53 to 1.90)
DR grade	
R1 (mild NPDR) [No. (%)]	34 (31.2)
R2 (moderate NPDR) [No. (%)]	29 (26.6)
R3 (severe NPDR) [No. (%)]	18 (16.5)
R4 (PDR) [No. (%)]	28 (25.7)
DME (yes) [No. (%)]	80 (73.4)

DR, diabetic retinopathy; SD, standard deviation; CI, confidence interval; BCVA, best-corrected visual acuity; DM, diabetes mellitus; NPDR, non-proliferative diabetic retinopathy; PDR, proliferative diabetic retinopathy; DME, diabetic macular edema.

### Utility values from patients and ophthalmologists

The TTO and RS utility values from the patients and ophthalmologists are shown in Table 2. The difference between the mean TTO utility values and RS utility values overall from patients was not statistically significant using the paired 2-tailed Student t test (*p* = 0.54). With the exception of differences in the means for Group 1 (Snellen BCVA of 20/20 to 20/25 in the better-seeing eye) (*p* <0.05), the differences between the mean utility values from the patients of each group using TTO versus RS methods were not statistically significant. However, with the exception of differences in the means for Group 4 (Snellen BCVA of 20/200 to 20/400 in the better-seeing eye) (*p* = 0.28) and Group 5 (Snellen BCVA worse than 20/400 in the better-seeing eye) (*p* = 0.30), the differences between the mean utility values as assessed by the ophthalmologists and each group using TTO and RS methods were statistically significant (*p* <0.05).

**Table 2:** Comparison of the time trade-off and rating scale utility values from patients and ophthalmologists.

Group	Visual acuity in the better-seeing eye.	No. (%)	Utility values assessed by patients			Utility values assessed by ophthalmologists		
			TTO, mean (SD), 95% CI	RS mean (SD), 95% CI	*p* Value*	TTO, mean (SD), 95% CI	RS mean (SD), 95% CI	*p* Value*
Overall	20/20 to worse than 20/400	109(100)	0.81 (0.10), 0.79 to 0.83	0.81 (0.11), 0.79 to 0.83	0.54	0.93 (0.07), 0.91 to 0.94	0.95 (0.04), 0.94 to 0.96	<0.05
1	20/20 to 20/25	59 (54.1)	0.84 (0.07), 0.82 to 0.86	0.86 (0.06), 0.84 to 0.88	<0.05	0.95 (0.04), 0.94 to 0.97	0.97 (0.02), 0.97 to 0.98	<0.05
2	20/30 to 20/50	37 (33.4)	0.81 (0.08), 0.78 to 0.83	0.77 (0.12), 0.73 to 0.81	0.07	0.93 (0.05), 0.91 to 0.94	0.94 (0.03), 0.93 to 0.95	<0.05
3	20/60 to 20/100	9 (8.3)	0.70 (0.10), 0.63 to 0.77	0.70 (0.06), 0.65 to 0.75	0.80	0.85 (0.08), 0.79 to 0.91	0.90 (0.05), 0.87 to 0.94	<0.05
4	20/200 to 20/400	2 (1.8)	0.55 (0.11), -0.41 to 1.50	0.73 (0.04), 0.41 to 1.04	0.32	0.74 (0.07), 0.10 to 1.38	0.91 (0.04), 0.53 to 1.29	0.28
5	worse than 20/400	2 (1.8)	0.56 (0.22), -1.41 to 2.52	0.60 (0.28), -1.94 to 3.14	0.50	0.72 (0.01), 0.59 to 0.85	0.76 (0.04), 0.37 to 1.14	0.30

TTO, time trade-off; RS, rating scale; SD, standard deviation; CI, confidence interval.

* *p* value comparing the TTO and SG methods within each visual group using the paired two-tailed Student’s t test.

### Disparities between the ophthalmologists and patients

**Fig 1:**
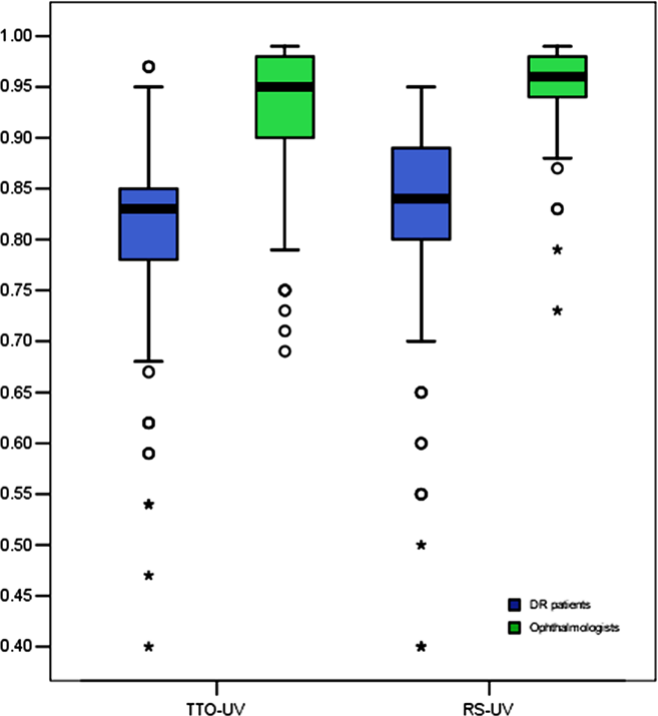
Distribution of utility values from diabetic retinopathy patients and ophthalmologists. The utility values were measured by time trade-off and rating scale methods. Boxes indicate the 25^th^ to 75^th^ percentiles of the utility values distribution, e.g., the interquartile range, and the bars inside the boxes represent the median. The whiskers extend to the lower and the upper extremes defined as 25^th^ percentile minus 1.5 times the interquartile range and the 75^th^ percentile plus 1.5 times the interquartile range. 〇, mild outliers; *, extreme outliers.

**Fig 2:**
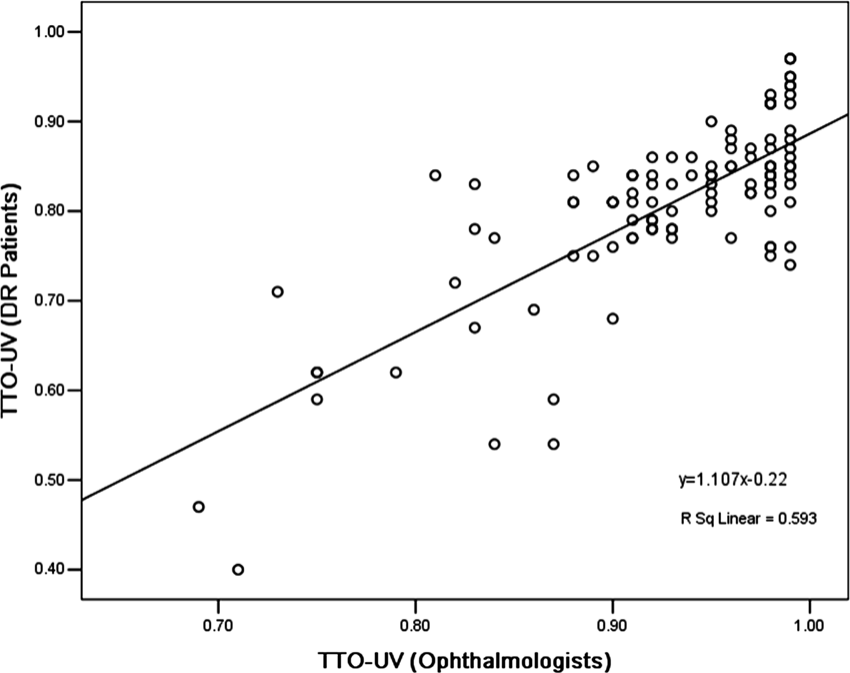
Scatter dot-plots of utility values from diabetic retinopathy patients and ophthalmologists, using the time trade-off method.

**Fig 3:**
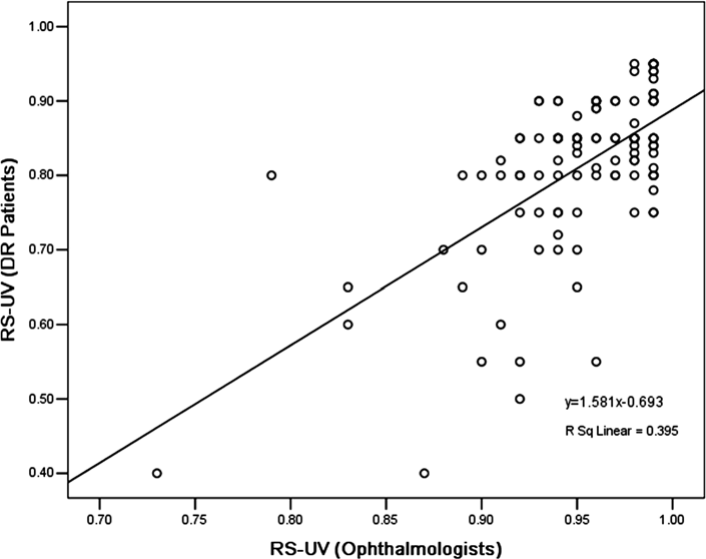
Scatter dot-plots of utility values from diabetic retinopathy patients and ophthalmologists, using the rating scale method.

We compared the difference in the mean utility values from the patients and ophthalmologists (Table 3). In addition to Group 4 (Snellen BCVA of 20/200 to 20/400 in the better-seeing eye) and Group 5 (Snellen BCVA worse than 20/400 in the better-seeing eye), the differences in the mean utility values between the patients’ and ophthalmologists’, overall, and each group using the paired 2-tailed Student t test were statistically significant (*p* <0.01) for both the TTO or RS methods. Actually, compared to the ophthalmologist preferences, the DR patient-assessed utility values were substantially lower than those of the ophthalmologists. Fig 1 shows box-plots for the utility values from DR patients and ophthalmologists, stratified by TTO and RS methods. In the total sample analysis, the utility values from DR patients and ophthalmologists had no significant correlations, for either the TTO (Fig 2) or RS (Fig 3) method.

**Table 3:** Comparison of the patients’ and ophthalmologists’ utility values using time trade-off and rating scale methods.

Group	Visual acuity in the better-seeing eye	TTO (t*, *p* *)	RS (t*, *p* *)
Overall	20/20 to worse than 20/400	-20.12, <0.01	-16.56, <0.01
1	20/20 to 20/25	-16.12, <0.01	-14.01, <0.01
2	20/30 to 20/50	-10.77, <0.01	-9.32, <0.01
3	20/60 to 20/100	-8,63, <0.01	-15.96, <0.01
4	20/200 to 20/400	-7.8, 0.08	-37.00, 0.02
5	worse than 20/400	-1.14, 0.46	-0.94, 0.52

TTO, time trade-off; RS, rating scale.

*t and *p* value comparing patients’ and ophthalmologists’ utility values within each visual group using the paired two-tailed Student’s t test.

For DR patients, multivariate analyses using linear regression for both TTO utility values and RS utility values as dependent variables are shown in Table 4 and Table 5, respectively. LogMAR BCVA in the affected eye and the average weighted LogMAR BCVA were the variables that significantly predicted both TTO utility values and RS utility values. In addition, DR grade and the duration of visual dysfunction were also variables that significantly predicted TTO utility values. Bivariate analyses were performed to determine which variables were independently associated with TTO utility values and RS, as shown in Table 6. Both TTO utility values and RS utility values were significantly associated with DME and employment status. Suffering from DME and unemployment were both factors independently associated with lower utility values.

**Table 4:** Predictors of time trade-off utility values from diabetic retinopathy patients and ophthalmologists, determined by multiple linear regression.

Predictor variable	Beta coefficient (95% CI)	*p* value*
TTO utility values		
(From DR patients)		
Constant	0.910 (0.871 to 0.948)	<0.01
LogMAR BCVA in affected eye	-0.093 (-0.158 to -0.028)	<0.01
Average weighted logMAR BCVA	-0.215 (-0.277 to -0.152)	<0.01
DR grade	0.180 (-0.003 to 0.038)	0.093
Duration of visual dysfunction	-0.013 (-0.026 to -0.001)	<0.05
(From ophthalmologists)		
Constant	0.994 (0.978 to 1.009)	<0.01
LogMAR BCVA in affected eye	-0.070 (-0.092 to -0.048)	<0.01
LogMAR BCVA in better eye	-0.120 (-0.151 to -0.089)	<0.01

TTO, time trade-off; CI, confidence interval; BCVA, best-corrected visual acuity; DR, diabetic retinopathy.

*Backward linear regression with *p* = 0.1 cut-off for exclusion was used.

**Table 5:** Predictors of rating scale utility values from diabetic retinopathy patients and ophthalmologists, determined by multiple linear regression.

Predictor variable	Beta coefficient (95% CI)	*p* value*
RS utility values		
(From DR patients)		
Constant	0.909 (0.879 to 0.939)	<0.01
LogMAR BCVA in affected eye	-0.076 (-0.131 to -0.021)	<0.01
Average weighted logMAR BCVA	-0.167 (-0.248 to -0.086)	<0.01
(From ophthalmologists)		
Constant	0.974 (0.956 to 0.992)	<0.01
LogMAR BCVA in affected eye	-0.037 (-0.050 to -0.024)	<0.01
LogMAR BCVA in better eye	-0.084 (-0.103 to -0.066)	<0.01
level of education	0.001 (0.000 to 0.002)	0.024

RS, rating scale; CI, confidence interval; BCVA, best-corrected visual acuity; DR, diabetic retinopathy.

*Backward linear regression with *p* = 0.1 cut-off for exclusion was used.

**Table 6:** Predictors of time trade-off and rating scale utility values from diabetic retinopathy patients and ophthalmologists, determined by bivariate analyses.

Predictor variable	Pearson Correlation	*p* value*
	(DR patients / Ophthalmologists)	(DR patients / Ophthalmologists)
TTO utility values		
DME	0.430 / 0.452	<0.01 / <0.01
Employment status	-0.266 / -0.200	<0.01 / <0.05
RS utility values		
DME	0.409 / 0.451	<0.01 / <0.01
Employment status	-0.204 / -0.219	<0.05 / <0.05

DR, diabetic retinopathy; TTO, time trade-off; RS, rating scale; DME, diabetic macular edema.

*Pearson correlation coefficients and analysis of variance (ANOVA) were used.

Furthermore, in the multivariate analysis of the utility values from ophthalmologists (Table 4 and Table 5), patients’ LogMAR BCVA in the affected eye and in the better eye were the variables that significantly predicted both the TTO utility values and RS utility values. In addition, patients’ education levels also significantly predicted RS utility values. In the bivariate analyses (Table 6), both TTO utility values and RS utility values were significantly associated with patients’ DME (*p* <0.01) and employment status (*p* <0.05), which was similar to the results from the patients.

### Comparison trade-off method with previous studies

We used one-way ANOVA to compare our TTO data with those of Brown and associates [23] obtained from 95 American patients with DR and from Sharma and associates [28] obtained from 186 Canadian patients with DR (Table 7). There was no difference in the mean utility values of the Chinese, American, and Canadian patients (F = 1.05, *p* = 0.35). With the exception of differences in the means for Group 3 (Snellen BCVA of 20/60 to 20/100 in the better-seeing eye) (F = 129.28, *p*<0.01), the differences among the mean utility values of each of the five groups stratified by categorical Snellen BCVA were not statistically significant. We simultaneously used the Dunnett t test to compare the difference between any two samples, and there were no cross border differences noted in the mean utility values, overall or for groups.

**Table 7:** Comparison of the time trade-off utility values from our sample with similar samples taken in the United States and Canada.

		TTO utility values, mean (SD)			
Group	Visual acuity in the better-seeing eye	Present sample (n = 109)	Brown et al. [23] (n = 95)	Sharma et al. [28] (n = 186)	Statistical value*
Overall	20/20 to worse than 20/400	0.81 (0.10)	0.77 (0.21)	0.79 (0.23)	F = 1.05, *p* = 0.35
1	20/20 to 20/25	0.84 (0.07)	0.85 (0.19)	0.881 (0.19)	F = 1.19, *p* = 0.31
2	20/30 to 20/50	0.81 (0.08)	0.78 (0.20)	0.786 (0.22)	F = 0.28, *p* = 0.76
3	20/60 to 20/100	0.70 (0.10)	0.78 (0.19)	0.728 (0.26)	F = 129.28, *p* <0.01
4	20/200 to 20/400	0.55 (0.11)	0.64 (0.15)	0.730 (0.22)	F = 1.05, *p* = 0.36
5	worse than 20/400	0.56 (0.22)	0.59 (0.37)	0.478 (0.47)	F = 0.07, *p* = 0.93

TTO, time trade-off.

*One-way ANOVA was used to calculate statistical values.

## Discussion

Because patient perspectives play an integral role in guiding important decisions, ophthalmologists are paying closer attention to the QoL of DR patients in the course of treatment. Although a number of visual-specific functioning and QoL questionnaires [11–16], and even DR-specific QoL questionnaires [31], have been developed and validated recently to measure the impact of DR-related QoL, our evaluation and understanding remain limited due to the restricted ability to fully assess QoL. Utility values were considered a useful tool to evaluate QoL associated with visual loss from the DR patient’s point of view [32,33] because this theoretically takes into account all aspects contributing to patient QoL and also provides an objective and comprehensive view of consequences [23,34]. Furthermore, it is much more convenient for the ophthalmologist to assess the patient’s QoL than to use visual-specific questionnaires composed of multidimensional items. We used two main types of utility measurements in our study: RS and TTO methods. The application shows that the RS method is an effective, simple and intuitive way to evaluate utility values. However, Cunningham and Hunt [35] suggested that the RS method should be used in conjunction with one of the other methods (SG or TTO) because it did not elicit valid cardinal utility measures. The TTO method has proven reliability, validity, and reproducibility and is widely used in ophthalmic research [36,37]. Although the SG method has been applied in many previous studies, we found it to be more difficult for patients to understand compared to the TTO and RS methods; in fact, some patients were intimidated by the concept of immediate death, no matter how small the chance [23]. Therefore, both the TTO and RS methods, which were used in our study rather than the SG method, are more readily understood by patients, especially older patients.

For the present sample of 109 DR patients, the mean overall TTO or RS utility values were 0.81. To exemplify the TTO theory, if a patient’s expected remaining lifespan was 20 years and he was willing to trade off 3.8 years for perfect vision, as previously described, the patient's score in rating his current vision was 81, in a range of 1 to 100. This means that the overall TTO utility value of DR patients in our study was slightly higher than the utility values of renal transplantation patients (0.78) [38], which was equivalent to age-related macular degeneration (0.81) [39] and was very similar to the results of other previous studies [27,28,40,41]. Nevertheless, we noted a considerable variance, ranging from 0.55 to 0.84, in the mean TTO utility values when the patients were grouped according to the severity of visual impairment in the better-seeing eye. In particular, utility values of DR patients with Snellen BCVA in the better-seeing eye of less than or equal to 20/200 (legal blindness in the USA) or less than 20/400 (legal blindness in China), were just slightly higher than the utility values of severe angina (0.5) [18].

A discrepancy between the mean RS utility values and the mean TTO utility values existed, especially in the low vision groups (Groups 4 and 5). This visual impairment may have the greatest effect on DR patients’ RS utility values. Therefore, the RS method reflected the patients’ subjective evaluation of their own vision status. TTO utility values may contain more variables that affect the QoL of DR patients, such as the degree of visual impairment, overall health status, family support, economic situation and cultural background. Therefore, the TTO method reflected individual differences in disease-related overall QoL. However, these two methods had a positive correlation (r = 0.707). In short, our results demonstrate that both TTO and RS utility values from DR patients showed good construct validity, as they were highly dependent on the degree of visual loss, whether in the affected eye or the average weighted LogMAR BCVA (Table 4), which is in accordance with conclusions from previous studies [23,28,40]. Thus, the greater is the degree of visual loss, the lower the mean utility values. In addition, DR patients’ TTO utility values were linearly correlated with the DR grade and duration of visual dysfunction. DR is mostly asymptomatic in its non-proliferative diabetic retinopathy (NPDR) stages but may cause significant and disabling vision loss once it progresses to severe NPDR and proliferative diabetic retinopathy (PDR) stages. There is an assumption that one could better adjust to visual loss over time [23]; thus, the utility values with chronic visual loss compared with more acute visual loss might be diverse. However, the conclusion from our study obviously did not prove this hypothesis. Those who had visual loss for a longer time were more willing to trade time for visual return. Furthermore, the level of education appeared to affect the mean utility values using the t test; nevertheless, multivariate regression analysis failed to confirm this association. Those with a high school education or less had lower utility values than those with formal education beyond the high school years, which is consistent with the results of a previous study [23]. We suspect that this discrepancy might be the result of individual economic and social status differences. It is worth mentioning that patients with substantially greater numbers of systemic comorbidities had similar utility values compared to those with no or minimal comorbidities. This result revealed that visual impairment is an independent and important factor impacting the QoL of DR patients.

DME and employment status also affected both the RS and TTO utility values, from both patients and ophthalmologists. DME that causes centralized vision loss was obviously associated with a negative impact on QoL [42]. However, in contrast to earlier views [28], unemployed patients had lower utility values than those who were employed. It is generally assumed that employed people require higher levels of visual function in order to perform better on the job. We suspected that this discrepancy might be a result of the limited sample size, and thus, we cannot be certain whether employment status was a significant confounder.

When presented with the scenario of visual loss secondary to DR, ophthalmologists substantially underestimated its effect on patients’ QoL. In our previous study [43] on rhegmatogenous retinal detachment and Brown’s study for AMD [44], differences between ophthalmologists’ and patients’ perceptions of QoL were observed. Ophthalmologists, who are usually concerned with the patient's visual acuity, the severity of the disease, and disease progression, ignored the psychological burdens on the patient from the disease itself, such as fear of blindness, the duration of visual dysfunction, and its impact on their daily life and work. This perspective has been confirmed by an analysis and comparison of the various factors that contribute to the patients’ experience of reduced QoL and the ratings provided by the ophthalmologists. This observation reinforces the importance of considering DR patients' perspectives and values when making significant health care decisions. At the focused level of individual patient care, this indicates that patients should play a significant role in decisions involving their treatment; at the broader level of health care policymaking, patients' preferences should help determine how limited resources are allocated.

Our conclusions, based on the above results, were basically consistent with results from other studies. Our hypotheses of how patients’ education levels and employment statuses significantly predicted utility values are as follows: patients differed in the extent of visual impairment; the number of DR patients with low vision was too small; our patients’ Snellen BCVA in the better-seeing eye was not lower than counting fingers, while other studies included patients with no light perception; and differences in social, economic, and cultural background.

As with any study, the present study has limitations. Although the sample size of our study was sufficient to calculate the overall utility values of DR patients, the numbers of patients in each group stratified by visual acuity were not sufficient. The utility values of DR patients should be measured by repeated questions at a later date because it is important to prove the reliability and repeatability of the results. Furthermore, the measurement of utility values, whether by TTO or RS, could have its own limitations, such as a ceiling effect. Furthermore, we were not able to take into account all variables that may affect the QoL of DR patients; it is possible that the utility values of patients with DR reflect patient suffering from diabetes systemically as a whole rather than DR alone. There is a danger that diabetes systemically acts as a confounding factor of the relationship between DR and utility values.

## Conclusions

Our data strongly suggest that DR causes a substantial decrease in Chinese patients’ utility values, which appears to be highly dependent on the degree of visual loss, and that ophthalmologists substantially underestimated its effect on patients’ QoL. Compared with previous studies in different countries, our conclusions were fundamentally similar.

## Supporting Information

S1 Text STROBE Statement—Checklist of items that should be included in reports of cross-sectional studies (DOC)

S2 Text PLOS ONE Clinical Studies Checklist. (DOCX)
